# Independent selection of eye and hand targets suggests effector-specific attentional mechanisms

**DOI:** 10.1038/s41598-018-27723-4

**Published:** 2018-06-21

**Authors:** Nina M. Hanning, David Aagten-Murphy, Heiner Deubel

**Affiliations:** 1 Graduate School of Systemic Neurosciences Department Biologie, Ludwig-Maximilians-Universität München, München, Germany; 2 Allgemeine und Experimentelle Psychologie, Department Psychologie, Ludwig-Maximilians-Universität München, München, Germany; 3 Department of Psychology, University of Cambridge, Cambridge, UK

## Abstract

Both eye and hand movements bind visual attention to their target locations during movement preparation. However, it remains contentious whether eye and hand targets are selected jointly by a single selection system, or individually by independent systems. To unravel the controversy, we investigated the deployment of visual attention – a proxy of motor target selection – in coordinated eye-hand movements. Results show that attention builds up in parallel both at the eye and the hand target. Importantly, the allocation of attention to one effector’s motor target was not affected by the concurrent preparation of the other effector’s movement at any time during movement preparation. This demonstrates that eye and hand targets are represented in separate, effector-specific maps of action-relevant locations. The eye-hand synchronisation that is frequently observed on the behavioral level must emerge from mutual influences of the two effector systems at later, post-attentional processing stages.

## Introduction

Previous research has shown that eye movements^1–3^ as well as hand movements^4,5^ are preceded by shifts of attention to their motor targets prior to movement onset. In everyday life, the control of these two movement types does not seem to be independent: When we interact with objects in our environment, our eye and hand movements frequently are highly coupled, both spatially and temporally. This raises the question whether eye and hand movements are attentional selected in unison, by one common mechanism, or whether they are selected individually by independent attention systems. Because of the observed interplay between both motor systems, the view that a shared, effector-agonist system underlies the selection of both eye and hand motor targets has gained wide support in numerous behavioral studies^6–11^. However, there is also psychophysiological evidence for the alternative view that eye and hand movements are selected by separate, largely independent attentional mechanisms^12^.

To resolve the ambiguity, we investigated the deployment of visual attention – an index of motor target selection – during the simultaneous preparation of saccadic eye movements and hand movements. In dual movement tasks we asked our participants to either reach or look, or to simultaneously reach and look towards certain target locations, while they concurrently discriminated the orientation of briefly presented oriented patterns, embedded in noise. We took the perceptual discrimination performance at motor targets and movement irrelevant locations as a measure of the distribution of attention during motor target selection. Critically, saccade and reach movement could be either directed to the same target location, letting us examine cumulative benefits, or to different locations, allowing the detection of an attentional trade-off between the eye and the hand motor target.

Our data reveals that during the process of motor preparation attention builds up in parallel at the saccade and the reach target. Importantly, the temporal dynamics of the shift of attention to both the saccade and the reach target do not differ between single (eye or hand) and combined (eye plus hand) movements. As the different action selection mechanisms did not compete for attentional resources at any time during movement preparation, our findings demonstrate that separate, effector-specific attentional mechanisms are responsible for selecting the motor targets for eye and hand movements.

## Results

### Attentional selection in single and combined eye-hand movements

**Figure 1:**
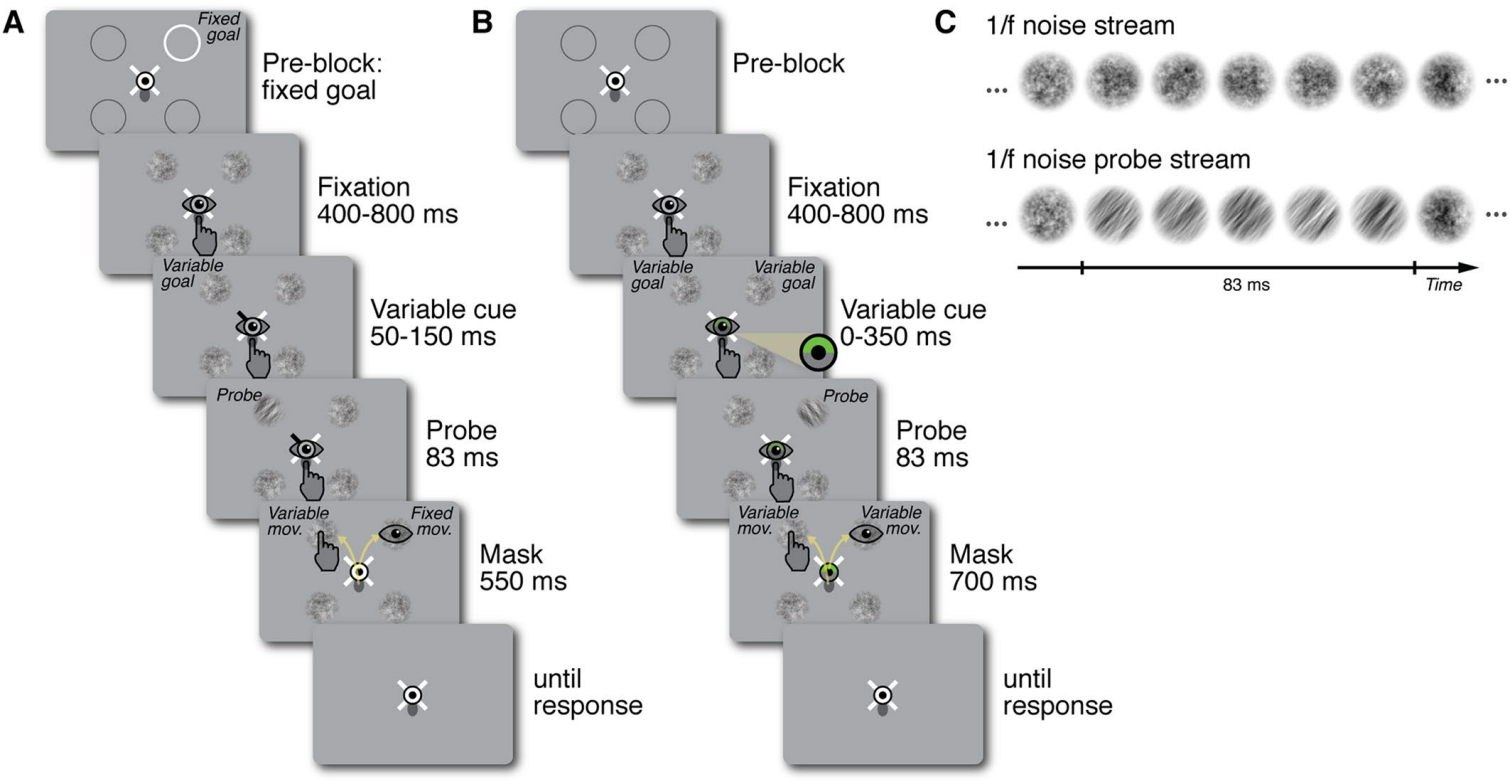
Design and stimuli. (**A**) Experiment 1, example motor task *Saccade*_*Fix*_*-Reach*_*Var*_: At the beginning of the block, the fixed motor target was marked by a white circle. Participants (n = 9) maintained eye and finger fixation until one of the four white direction lines pointing towards the noise patches turned black, indicating the variable motor target. Participants reached towards the variable motor target and simultaneously saccaded towards the fixed motor target. 50–150 ms after cue onset one of the noise streams showed a clockwise or counterclockwise orientation. After a masking period, participants indicated their discrimination judgement via button press. (**B**) Experiment 2, example motor task *Combined*: Participants (*n* = 9) maintained eye and finger fixation until half of the eye fixation target turned green, revealing two potential motor targets (here the two upper locations). Participants reached towards one of the two potential motor targets and simultaneously saccaded towards the other. 0–350 ms after cue onset, one of the noise streams showed a clockwise or counterclockwise orientation. After a masking period, participants indicated their discrimination judgement via button press. See also Supplementary Movie S1 (**C**) Noise streams used as discrimination stimuli. Each of the four noise streams consisted of a succession of randomly generated 1/f noise patches, flipping at 60 Hz. The probe stream comprised a 83 ms sequence of orientation filtered 1/f noise patches, showing a 40° clockwise or counterclockwise orientation.

In Experiment 1 (Fig. 1A) participants performed single or combined movements. In the single motor tasks, they made an eye or hand movement towards either a fixed (*Saccade*_*Fix*_ or *Reach*_*Fix*_) or a variable target (*Saccade*_*Var*_ or *Reach*_*Var*_), while their other effector remained at fixation. In the combined motor tasks, they executed a combined eye-hand movement. One effector was consistently moved towards a fixed target, the other effector’s target varied on each trial (*Saccade*_*Fix*_*-Reach*_*Var*_ or *Saccade*_*Var*_-*Reach*_*Fix*_). While the location of the variable target was unpredictable and cued centrally on each trial, the fixed target was indicated at the beginning and remained the same throughout the experimental block. The four possible motor targets were indicated by streams of 1/f noise patches separated by 90° and positioned 8° from the fixation. Participants were instructed to initiate their movement(s) as soon as the cue appeared. 50–150 ms after cue onset (within the movement latency) an orientation discrimination probe appeared at one of the four locations with equal likelihood (Fig. 1C), meaning that it could occur either at a motor target or at a non-target location. After presentation of the orientated probe, the stream of noise patches continued (acting as a mask) for another 316.6–416.6 ms, after which participants indicated whether the perceived orientation of the probe was tilted clockwise or counterclockwise.

**Figure 2:**
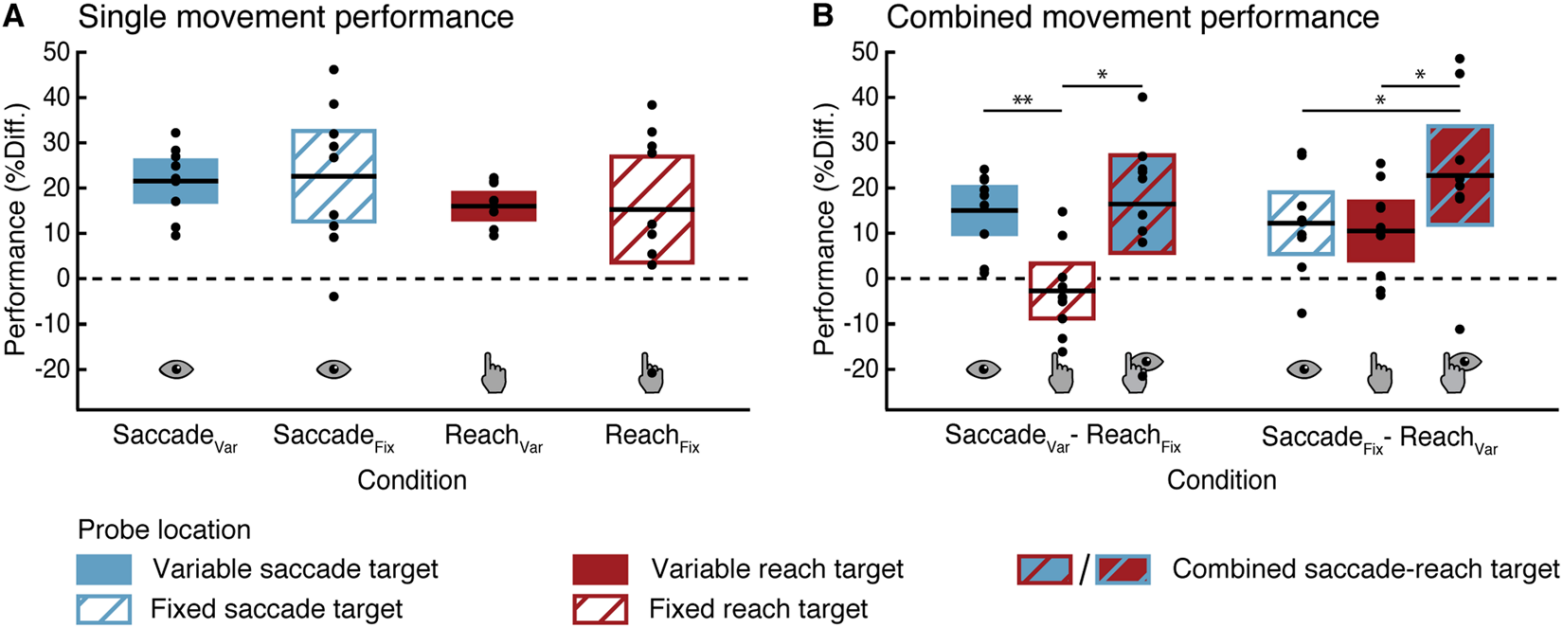
Attentional selection in single and combined movements in Experiment 1. Discrimination performance for the single eye or hand movement tasks (**A**) and the combined eye-hand movement tasks (**B**). Black lines within each whisker plot indicate the average difference in discrimination performance between each condition’s motor target(s) minus the respective baseline performance at non-target locations. Coloured/striped bars show the 95% confidence interval. Dots represent individual subject data. Horizontal dashed lines mark non-target baseline performance. *p < 0.05, **p < 0.01, significant difference between two motor targets.

To demonstrate that attention was allocated to each effector’s target before movement onset, we first analyzed discrimination performance in the single motor tasks (Fig. 2A). All comparisons were contrasted to the non-target locations unless otherwise stated. We found that performance at the saccade target was significantly enhanced (*p* < 0.01), regardless of whether the target location varied from trial-to-trial (*Saccade*_*Var*_; *d* = 2.59), or was consistent across the block (*Saccade*_*Fix*_; *d* = 1.66). Similarly, discrimination performance at the variable reach target (*Reach*_*Var*_; *d* = 1.50) was significantly improved (*p* < 0.01). However, when participants consistently reached towards the same location (*Reach*_*Fix*_), discrimination performance was the same as at non-target locations (*P* > 0.05).

When performing combined motor tasks, we found that for a fixed reach and a variable saccade (*Saccade*_*Var*_*-Reach*_*Fix*_; Fig. 2B, left), discrimination performance increased only at the variable saccade target (*p* < 0.01, *d* = 1.53). Performance at the fixed reach target, however, was no different than at non-target locations (*p* > 0.05). While there was a significant enhancement in discrimination performance when the goals of variable saccade and fixed reach coincided (*p* < 0.05, *d* = 1.23), this combined target performance did not exceed that at a spatially separate variable saccade target, when the reach was directed elsewhere (*p* > 0.05). Hence, when the reach target was fixed throughout a block, attention was no longer allocated to that location.

In contrast, when participants reached towards variable targets while making eye movements to a fixed location (*Saccade*_*Fix*_*-Reach*_*Var*_; Fig. 2B, right), we observed a strikingly different pattern: Discrimination performance at both the saccade (*d* = 1.20) and the reach target (*d* = 0.87) was significantly enhanced (*p* < 0.05). Importantly, when performing a combined movement to different locations, performance at the saccade (or reach) target location was approximately the same as when participants made only a single saccade (or reach). This suggests that selection mechanisms for the individual effectors operated independently and in parallel, such that the selection of one effector’s motor target did not affect the attentional selection of the other.

Furthermore, when variable reach and fixed saccade target coincided, performance at this common motor target was enhanced (*p* < 0.01, *d* = 1.70), and critically it significantly exceeded the performance observed at a spatially separate fixed saccade target (*Saccade*_*Fix*_*; p* < 0.05, *d* = 0.77), and at a separate variable reach target (*Reach*_*Var*_; *p* < 0.01, *d* = 0.77). This suggests that saccade and reach target selection acted synergistically when both movements were directed towards a common goal, leading to a greater enhancement than would be expected from either movement alone.

### Attentional dynamics of eye and hand target selection

**Figure 3:**
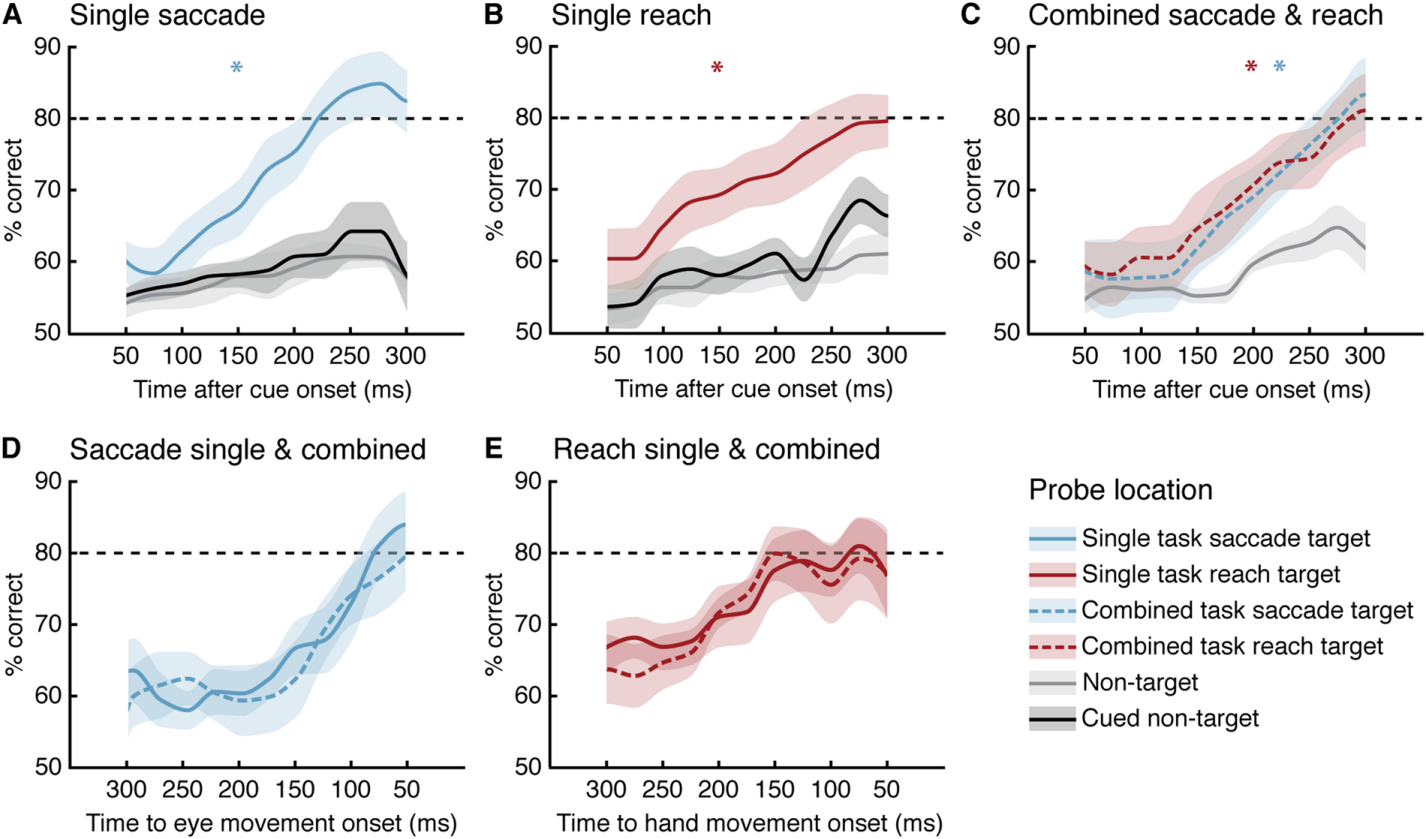
Temporal dynamics of attentional selection in single and combined movements in Experiment 2. (**A**,**B**) Discrimination performance in the single eye or hand movement tasks as a function of cue-onset to test-offset at the saccade target (blue), at the reach target (red), at the unchosen cued location (black) or at non-target locations (gray). (**C**) Performance in the combined movement task as a function of cue-onset to test-offset at the saccade target (dashed blue), at the reach target (dashed red), or at non-target locations (gray). (**D**,**E**) Performance as a function of test-offset to movement onset at the single saccade target (blue) vs. combined saccade target (dashed blue) and single reach target (red) vs. combined reach target (dashed red). Data are represented as mean, coloured areas denote the standard errors of the mean. *p < 0.05, earliest significant difference between motor target (coloured) and non-target locations (gray).

To examine the degree to which saccade and reach selection were dynamically independent, Experiment 2 investigated how attentional deployment to the different motor targets developed over time. Since the results of Experiment 1 suggested that participants might utilise different attentional strategies when the reach target is fixed, both the saccade and the reach varied in Experiment 2 (Fig. 1B, see also Supplementary Movie S1). Participants either made a single movement to one of two centrally cued targets (*Saccade* or *Reach*), or performed a combined eye-hand movement (*Combined*), directing one effector to each of the cued locations at free choice. At various time points after cue onset (0–350 ms; within the movement latency), an orientation discrimination probe was presented randomly at one of the four locations. We computed the mean discrimination performance at different times after cue presentation, using a 100 ms moving average, stepping every 25 ms from test presentation 50 to 300 ms after cue onset (Fig. 3A–C).

In the single movement tasks, discrimination performance at the selected saccade or reach target increased gradually over time, while it remained near chance at the unselected location and at the non-target locations. In the *Saccade* task (Fig. 3A) discrimination performance at the saccade target was significantly superior to all remaining locations approximately 150 ms after cue onset (*p* < 0.05, *d* = 1.07). Likewise, in the *Reach* task (Fig. 3B) after 150 ms performance at the reach target was significantly better than non-target performance (*p* < 0.05, *d* = 1.22). Remarkably, in the *Combined* task performance increased simultaneously at both motor targets (Fig. 3C, and did not differ between the two locations at any point in time (*P* > 0.05), indicating that attention was dynamically allocated to both targets in parallel.

Next, we examined whether attentional dynamics differed between single and combined movements. To account for latency differences, trials were binned as a function of the time between discrimination probe offset and movement onset. Performance at the saccade target was strikingly similar throughout the entire period, regardless of whether participants were required to make a single saccade or a combined saccade and reach (*Saccade vs Combined; P* > 0.05, Fig. 3D). Likewise, performance at the reach target developed independently of whether participants performed only a reach or made an additional saccade *(Reach vs Combined; P* > 0.05, Fig. 3E).

## Discussion

In this study we not only resolve a long-standing controversy about whether eye and hand targets are selected by a unitary or by independent attentional systems, but also identify some of the reasons why previous studies have produced such divergent results. By asking our participants to perform simultaneous eye-hand movements to separate locations, we demonstrated that attention builds up at both the saccade and reach target in parallel, without any trade-off between the two motor targets. Importantly, we found no evidence that the selection mechanisms for the different effectors compete for attentional resources at any time during movement preparation, and demonstrate that eye and hand targets are represented in independent, effector-specific maps of relevant locations.

Previous studies^6,12^ investigating whether different effectors are related to separate target selection mechanisms have produced mixed results, although they utilized quite similar protocols. However, our more extensive design allowed us to not only replicate their findings, but also to resolve the ambiguity in their interpretation. In accordance with earlier work^12^, we observed that in combined eye-hand movements attention was allocated in parallel to both the fixed saccade and the variable reach target, with attentional performance at each target being approximately equivalent to the respective single movement condition. Moreover, we also observed a synergistic effect when hand and eye were directed towards the same location, suggesting that planning the combined movement of two effectors recruited more attentional resources than when only a single effector was engaged. Overall, our results support the previously employed broad conclusion, that separate effector-specific attentional mechanisms are responsible for the selection of eye and hand targets^12^.

However, while the aforementioned study^12^ investigated attention with a fixed eye target and a variable reach target, another study^6^ also examined the inverse (fixed reach and variable saccade). They observed no benefit at the fixed reach target when a simultaneous saccade to a variable, different target location co-occurred, which they interpreted as evidence that the eye dominates attention in combined eye-hand movements. Yet, as we found in our first experiment, the repeated execution of a reach movement to a fixed location – even without the competition of a simultaneous saccade – did not yield a shift of attention towards the reach target. Presumably, repeated hand movements, similar to delayed hand movements^13^, can be preprogrammed and therefore are not preceded by the typically reported shift of attention to the motor target^1–3^. Accordingly, our results suggest that the alleged dominance of the eye in guiding visual attention during simultaneous eye-hand movements^6^ may instead be an artefact of repetitive hand movements to a fixed location ceasing to recruit reach-related attentional resources.

Our results demonstrate a difference in the premotor allocation of attention depending on the type of movement: while variably cued movements inevitably draw attention to their motor target, repeatedly performed, fixed hand actions do not cause this compulsory shift of attention. Furthermore, a comparison of the single movement data of Experiment 1 revealed a larger between-subject variance in attentional performance at the targets of fixed compared to variable eye and hand movements (Fig. 2A). In the fixed movement conditions, as one location was task-relevant throughout a whole experimental block, some participants may have endogenously attended to that location, boosting their performance, suggesting that fixed, repetitive movements are vulnerable to attentional strategies.

When investigating how attention was dynamically allocated to the movement targets in Experiment 2, we therefore ensured that both the eye and the hand target were variable and unpredictable. Furthermore, presenting the test orientation at various time points after the motor cue allowed us to study the temporal dynamics with which hand- and eye-based attentional resources were allocated to different target locations. We observed that when variable eye-hand movements were performed simultaneously, attention increased gradually at the saccade and the reach target. Remarkably, the dynamics of the shift of attention towards each effector’s motor target did not depend at any time during movement preparation on whether or not a movement of the other effector was prepared simultaneously. As the gradual increase of discrimination performance at one effector’s motor target was not affected by the simultaneous preparation of a movement of the other, our results demonstrate that eye and hand movement targets are selected by independent, effector-specific attentional mechanisms.

This result seems at odds with the common observation that our eye and hand movements are highly coupled, both spatially and temporally, when we interact with objects in the environment. In free-viewing tasks the eyes systematically move to reach targets before hand movement onset^10,14–16^. Because of the observed interplay between both motor systems, many behavioral studies measuring various motor parameters such as movement precision, amplitudes, velocity profiles, and latencies, have favoured the view that one shared effector-agonist system underlies the selection of eye and hand movement targets^9–11^.

The interaction between the eye and the hand movement system at the behavioral level has a neurophysiological basis. Functional imaging studies in humans observed an overlap of parietal and pre-frontal cortical areas involved in eye and hand target selection^17,18^. In line with this, single-cell recording studies report that reach-related activity of neurons in the parietal cortex is modulated by eye position^19^, while the activity of neurons in the primarily oculomotor-related areas like the supplementary eye field (SEF) and the frontal eye field (FEF) is modulated by hand position signals^20,21^. Furthermore, saccadic representations in the lateral intraparietal area (LIP) are influenced by a simultaneous reach movement^22^, lesions of area LIP have been observed to delay the onset of reaches but only when they are accompanied by a saccade^23^, and coherent spiking of neurons in this area has been suggested to coordinate eye and hand movements^24^.

At first glance, the reported behavioral and neurophysiological dependencies between the two effector systems strongly argue in favour of a common, shared system serving the selection of eye and hand targets. Yet, our results demonstrate the opposite, namely that independent systems individually select eye and hand targets. We argue that the frequently reported cross-talk between the two motor systems presumably results from interactions at later processing stages, with the initial attentional selection of the motor targets being largely independent for eye and hand movements.

This conclusion is in line with imaging studies in humans which found that eye and hand movements are preceded by activity in separate parietal areas^25,26^. Furthermore, there is neurophysiological evidence that neural circuits responsible for the generation of eye and hand movements are implemented by functionally and anatomically distinct brain areas. While the brain circuit dedicated to eye movements comprises FEF and LIP^27–31^, the circuit serving the production of hand movements involves more dorsal areas of the promotor cortex (PMd) and the parietal reach region (PRR) e.g^31–34^. These effector-specific neural circuits conceivably can give rise to the independent shifts of attention that we observed at the targets of eye and hand movements.

The neural mechanisms that guide the allocation of attention to salient or action-relevant locations have been referred to in the recent literature as priority maps^35,36^. In line with our assumption of separate attentional mechanisms, there is evidence that saccade and reach preparation may rely on different priority maps: While FEF and LIP are involved in saccade preparation, the PRR might code the behavioral priority for reach movements^31,37^. If a stimulus becomes relevant for a given effector, activity at the corresponding location in the respective priority map increases and triggers a feedback signal to earlier visual areas^38^. At the subcortical level, the superior colliculus (SC), for instance, receives projections from oculomotor as well as from hand-related areas and reportedly encodes priority irrespective of the effector^39–41^. The above described interaction between the eye and the hand motor system on the behavioral level, including the synergistic effect observed in our first experiment, which occurs when eye and hand movement are directed towards one common motor target, also reported in^12^, can be explained by such feedback connections within saccade- and reach-related circuits converging onto earlier visual areas^42,43^.

In conclusion, our findings demonstrate that motor preparation in the eye and the hand movement systems produces independent shifts of attention, suggesting that saccades and reaches are represented in separate, effector-specific maps of action-relevant locations. While previous findings have been interpreted in favour of a coupled model, or one in which a specific effector dominates the other, we here show that reach and saccade target selection can be completely dissociated at the behavioral level. Indeed, even though the frequently observed yoking of eye and hand movements indicates that eye-hand coupling is beneficial, this does not imply it is mandatory. Independent systems – which can be coupled as required – enable individual targeting when necessary, e.g. to accomplish complex tasks such as tool use and bimanual manipulation.

## Methods

### Subjects and apparatus

Nine right handed human observers (5 females, ages 23–28 yr, one author) completed Experiment 1. Nine right handed human observers (eight of whom also participated in Experiment 1; 5 females, ages 23–28 yr, one author) completed Experiment 2. All participants gave informed consent. The protocols for the study were approved by the ethical review board of the Faculty of Psychology and Education of the Ludwig-Maximilians-Universität München, in accordance with German regulations and the Declaration of Helsinki. Gaze position was recorded using an EyeLink 1000 Tower Mount (SR Research, Osgoode, Ontario, Canada) at a sampling rate of 1 kHz. The experimental software was implemented in Matlab (MathWorks, Natick, MA, USA), using the Psychophysics^44,45^ and EyeLink toolboxes^46^. Stimuli were presented on a 45° inclined touchscreen (Elo 2700 IntelliTouch, Elo Touchsystems, Menlo Park, CA) with a spatial resolution of 1280 × 1024 pixels and a refresh rate of 60 Hz.

### Procedure and Stimuli

#### Experiment 1

In this experiment we investigated the deployment of visual attention in single eye, single hand and coordinated eye-hand movements, that were directed towards fixed or varying target locations. We measured attentional distribution during motor target selection by comparing orientation discrimination performance at single and combined motor targets with performance at non-target locations.

In a randomised block design, participants performed single eye movements, single hand movements, or combined eye-hand movements. In the single movement blocks, the motor target either was fixed, i.e. the eye movement was repeatedly executed towards the same location, or varied randomly between trials. Combined eye-hand movement blocks consisted of one fixed and one variable motor target. The experiment comprised six motor tasks: variable eye movement (*Saccade*_*Var*_), variable hand movement (*Reach*_*Var*_), fixed eye movement (*Saccade*_*Fix*_), fixed hand movement (*Reach*_*Fix*_), fixed eye & variable hand movement (*Saccade*_*Fix*_-Reach_Var_), and fixed hand & variable eye movement (Saccade_Var_-*Reach*_*Fix*_). Figure 1A depicts the sequence for the *Saccade*_*Fix*_-Reach_Var_ task: Participants initially fixated a central fixation target comprising a black and white bull’s eye (0.5° radius) on a uniform gray background. Their right index finger remained on a gray oval (0.6° × 0.65°) slightly below the eye fixation. At the beginning of each block, four equally spaced locations were marked by gray circles (2° radius) 8° away from fixation, with four white direction lines (0.1°-width, 0.4°-length) surrounding fixation, pointing towards them. One of the four locations (randomly selected) was framed in white, indicating the fixed motor target. Participants memorized this location, as it would constitute their saccade target throughout the whole block. Once stable eye and finger fixation was detected within a 2.5° radius virtual circle centered on the fixation targets, four streams of 1/f noise patches (2° radius) appeared at the marked locations. Each noise stream consisted of randomly generated 1/f noise patches windowed by a symmetrical raised cosine (radius 2°, sigma 0.5), flipping at 60 Hz (Fig. 1C). After a delay of 400–800 ms, one of the direction lines turned black, indicating the variable motor target. The location was selected randomly and could coincide with the fixed motor target. The onset of the line cue was the go-signal for both movements. Participants reached as fast and as precise as possible to the noise stream corresponding to the black line (reach target) and simultaneously saccaded as fast and precise as possible to the fixed saccade target cued in the beginning of the block. 50–150 ms after cue onset (within the movement latency), one of the 1/f noise streams was briefly replaced by an orientation-filtered noise stimulus, showing a 40° clockwise or counterclockwise orientation. This test signal was equally likely to appear at any of the four locations and was masked by the reappearance of non-oriented 1/f noise after 83 ms. After another 1000 ms the screen turned blank and participants indicated via button press in a non-speeded manner whether they had perceived the orientation to be tilted clockwise or counterclockwise, receiving auditory negative feedback for incorrect responses.

All other motor tasks had the same timing and stimuli, but differed in cueing procedure and pre-block instruction: In the *Saccade*_*Var*_-*Reach*_*Fix*_ task, participants were instructed to reach towards the fixed target and saccaded according to the variable cue. In the variable single movement tasks, no fixed target was marked at the beginning of the block and participants made a saccade or a reach according to the variable line cue, while keeping fixation with the other effector. In the fixed single movement tasks, instead of one line marking the variable target all lines turned black, functioning as the go signal for the single fixed eye or hand movement. The other effector remained at fixation.

Participants performed 12 experimental blocks (4 single, 8 combined-movement) of at least 140 trials each. We controlled online for broken eye and finger fixation (outside 2.5° from fixation), too short (<100 ms) or too long (>500 ms) movement latencies, and incorrect eye or hand movements (not landing within 2.5° from target). Erroneous trials were repeated in random order at the end of each block. Overall, participants made eye movement errors in 14.8 ± 2.3 (mean ± SE) % and finger movement errors in 17.9 ± 2.8% of the trials. To maintain a consistent level of discrimination difficulty across participants, a threshold task preceded the experiment, in which we determined the individual orientation filter strength (i.e. the visibility of the tilt) corresponding to 80% correct discrimination. The threshold task matched the main experiment, but with participants maintaining eye and finger fixation and the discrimination signal predictably appearing always at the fixed cued location.

#### Experiment 2

In Experiment 2 we investigated how attentional deployment to the different motor targets developed over time. We asked participants to execute single eye or hand, or combined eye-hand movements towards two variable and simultaneously cued target locations, and presented the test orientation at various time points after the motor cue.

The experiment comprised three motor tasks: variable eye movement (*Saccade*), variable reach movement (*Reach*), and variable eye - hand movement (*Combined*). The motor tasks had the same timing and stimuli, and differed only in the pre-block instruction. Figure 1B depicts the sequence for the *Combined* task, that was identical to Experiment 1, except for the following differences: (1) No fixed motor target was marked at the beginning of the block. (2) The variable motor targets were indicated by half of the eye fixation turning green (the left, right, upper, or lower half), revealing two potential motor targets (the two left, right, upper, or lower locations, respectively). Participants reached towards either of the two potential motor targets while simultaneously making a saccade towards the other – at free choice. In the *Saccade* and *Reach* tasks, participants were instructed to make only one movement (saccade or reach, respectively) to either of the potential motor targets – again at free choice –, while keeping fixation with the other effector. The onset of the colour cue also functioned as the go signal for the movement(s). (3) The discrimination orientation within of the 1/f noise streams appeared after a variable delay of 0–350 ms after cue onset. For a demonstration of the trial sequence see also Supplementary Movie S1.

Participants performed 12 experimental blocks (three of each motor task, beginning with the single movement tasks in random order) of at least 160 trials each. Overall, participants made eye movement errors in 12.5 ± 1.4 (mean ± SE) % and finger movement errors in 14.6 ± 1.73% of trials, which were repeated in random order at the end of each block.

### Data analysis

In both Experiments we detected saccades offline based on the eye velocity distribution^47^. We measured finger movement onset and landing time, as well as landing position. We accepted trials (1) if eye and finger fixation were maintained within 2.5° from the fixation until cue onset, (2) if movement latencies were no shorter than 100 ms and no longer than 500 ms, (3) if the saccade and/or reach landed within 2.5° from the cued target, (4) if the passive effector maintained fixation in the single movement tasks, (4) if no blink occurred during the trial, and (5) neither eye nor finger movement started before the offset of the test orientation. We took the average percentage correct orientation discrimination performance (clockwise or counterclockwise) at the four tested locations as a proxy of attentional selection. In Experiment 1 we calculated difference scores by subtracting the respective performance at non-target locations from each motor target performance. For statistical comparisons we resampled our data and derived p-values by locating any observed difference on the permutation distribution (difference in means based on 1000 permutation resamples). For the time course analysis of Experiment 2, we binned the data separately for the different locations according to the SOA between motor cue onset and orientation discrimination probe offset (from 0 to 350 ms) in 11 time bins by using a 100 ms sliding time window stepping every 25 ms (first time bin 0 to 100 ms SOA, second time bin 25 to 125 ms SOA etc.). For each location, we then computed the average discrimination performance for each time bin and interpolated the data for the visualization in Fig. 3.

### Data availability

The datasets generated during and/or analysed during the current study are available from the corresponding author on reasonable request.

## Electronic supplementary material

Supplementary movie

Supplementary Information
