# Supplementary material for: Independent selection of eye and hand targets suggests effector-specific attentional mechanisms
Source: Sci Rep. 2018 Jun 21;8:9434. doi: 10.1038/s41598-018-27723-4 (PMC6013452; doi:10.1038/s41598-018-27723-4)
Supplement: Supplementary file 2 — Supplementary Information [file 41598_2018_27723_MOESM2_ESM.pdf]

Supplementary Information

# Independent selection of eye and hand targets suggests effector-specific attentional mechanisms

Nina M. Hanning[1,2*], David, Aagten-Murphy[3] & Heiner Deubel[2]

1. *Graduate School of Systemic Neurosciences*
   *Department Biologie, Ludwig-Maximilians-Universität München, Germany.*
2. *Allgemeine und Experimentelle Psychologie,*
   *Department Psychologie, Ludwig-Maximilians-Universität München, Germany.*
3. *Department of Psychology,*
   *University of Cambridge, Cambridge, UK.*

***** corresponding author & Lead contact: Nina Hanning (hanning.nina@gmail.com)
Leopoldstr 13, 80802 München, Germany

Supplementary Information

**Legend**

**Movie S1.** Related to Figure 1B. Demonstration of the trial sequence of Experiment 2. Click on the video to start. Look at the central fixation bull's eye and place your index finger at the grey oval beneath. Half of the bull's eye will turn green, revealing two potential motor targets (e.g. if the upper half turns green, the upper two locations are the motor targets). For the *Combined* task, reach towards one of the two targets and simultaneously look towards the other. At the same time, try to discriminate the orientation (clockwise or counterclockwise) that will briefly appear at any of the four locations.
